# Frequency, combinations and clinical relevance of outpatient low back pain diagnoses: analysis of claims data in a population-based cohort study

**DOI:** 10.1186/s12891-025-08514-1

**Published:** 2025-04-03

**Authors:** Elisa Kasbohm, Jean-François Chenot, Carsten Oliver Schmidt, Julia Truthmann

**Affiliations:** 1 Department SHIP-KEF, Institute for Community Medicine, University Medicine Greifswald, 17475 Greifswald, Germany; 2 Department of General Practice, Institute for Community Medicine, University Medicine Greifswald, 17475 Greifswald, Germany

**Keywords:** Low back pain, Specific LBP, ICD-10 coding, Outpatient care, Claims data

## Abstract

**Background:**

Claims data are often used to investigate the quality of care for patients with low back pain (LBP). However, there is no standard regarding the preferred choice of ICD-10 codes for identifying patients with LBP, and guidelines for the treatment of LBP differ in their interpretation of ICD-10 codes. Furthermore, for some indicators measuring the quality of care, such as the appropriate use of imaging, it is necessary to differentiate between cases with specific, treatable causes and those without. This study therefore investigates coding practices for LBP in outpatient care and the use of imaging across specialist groups over a six-year period.

**Methods:**

Based on the TREND cohort of the population-based Study of Health in Pomerania (SHIP), coding practices in claims data were analysed using data from 3,837 statutorily insured participants for the years 2014–2019. In total, eleven ICD-10 categories of relevance to LBP were included. We evaluated the findings based on two German guidelines: one for specific and one for non-specific LBP.

**Results:**

At least one LBP diagnosis was coded for 2,474 participants (64%) during the entire observation period. The predominant ICD-10 category was M54 (dorsalgia, 87% of patients with LBP). Around half of the participants with M54 diagnoses also had diagnoses from other LBP-related categories in the same year. Diagnoses that can be assigned to specific LBP according to the respective German guideline occurred in 86% of patients with LBP. Participants who consulted only general practitioners during the observation period were more likely to receive only an M54 diagnosis and less likely to undergo imaging procedures.

**Conclusions:**

The results underline the high epidemiologic relevance of LBP. Using the German guideline on specific LBP as a reference, we categorized most LBP diagnoses as specific, contrary to common international assumptions. Most patients with LBP received multiple ICD-10 codes, complicating the distinction between non-specific and specific LBP based on claims data. Health care analyses on LBP require transparent reporting of the ICD codes used, along with a detailed discussion of the data’s limitations.

**Supplementary Information:**

The online version contains supplementary material available at 10.1186/s12891-025-08514-1.

## Background

Low back pain (LBP) is one of the most common and costly reasons for consultations in outpatient care, significantly impacting health economics due to frequent and prolonged sick leave [1, 2]. Improving patient care and optimising resource allocation is therefore highly relevant.

Claims data can provide valuable insights for this purpose. For example, the German quality indicator set ‘QISA’ (quality indicator system for outpatient care) [3, 4] measures structural and process quality of care based on ICD-10 coded diagnoses and fee schedule items. Many health insurance companies utilize claims data for health reporting purposes [5] and often make their data available for secondary analyses [6, 7]. In another example, the Global Burden of Disease Study utilizes claims data as well as survey data to estimate years lived with disability due to LBP and its current and future prevalence [8].

Despite the wide use of claims data to assess health care, considerable uncertainties remain. Health reports and published studies are not consistent regarding the selection of ICD-10 codes used to identify patients with LBP from claims data, which limits the comparability of data and the ability to monitor the effectiveness of health services [9]. In particular, determining whether a specific treatable cause exists or if general therapeutic measures are sufficient plays a crucial role in clinical decision making for LBP. The former is commonly referred to as specific LBP, while the latter is termed non-specific LBP. Appropriate differentiation is key to adequately managing patients with LBP, but guidelines differ considerably regarding their views on which ICD-10 codes should be used for specific LBP.

The German guideline on non-specific LBP [10] considers the ICD-10 classification unsuitable for distinguishing between specific and non-specific LBP. It advises against further diagnostic investigations at the initial stage of LBP if the patient’s history and clinical assessment do not indicate signs of serious disease (red flags) [10]. The focus is rather on active self-management strategies [10]. According to this view and in line with international guidelines, the assumption is that most cases of LBP are of non-specific origin and that a maximum of 5–15% of patients have specific LBP [11, 12].

In contrast, the German guideline on specific LBP takes an entirely different stance in explicitly linking a wide range of ICD-10 codes to specific LBP [13]. Examples for morphological entities linked to specific LBP by the guideline are lumbar facet syndrome / spondylarthrosis, discogenic lumbar syndrome, and vertebral osteochondrosis, among others (Table Z1) [13]. In the work-up for patients with specific LBP, this guideline recommends a detailed analysis of symptoms, laboratory tests and imaging [13]. Treatment is tailored to the underlying assumed cause identified by the diagnostic work-up. For example, according to the guideline, a diagnosis of ‘other spondylosis with radiculopathy’ justifies interventional procedures, including epidural or intradiscal injections, and may be an indication for surgical treatment [13].

Overall, the German guideline on specific LBP [13] suggests a wider usage of diagnostic imaging and, assuming more frequent specific causes of pain, recommends earlier initiations of medical and surgical treatments compared to the German guideline on non-specific LBP [10]. Even though the diagnosis of specific LBP usually requires imaging, the wide use of diagnostic imaging may lead to overdiagnosis and overtreatment [10]. The issue is that imaging procedures are highly sensitive for detecting morphological findings but not for identifying etiologically or clinically relevant conditions, which can sometimes lead to unnecessary invasive treatments [14]. An example of this are degenerative changes, such as spinal osteochondrosis, spondylosis, and other spondylopathies, which occur frequently in people without LBP as well, depending on their age [14]. Thus, the connection between symptoms of LBP and specific diagnoses based on radiological findings is generally considered uncertain [15].

Taken together, there exist some remarkable differences in the recommendations regarding the use of ICD-10 codes in the context of specific LBP. The extent to which coding practices for LBP vary across specialist groups and the impact of guideline recommendations regarding ICD-10 codes on claims data-based estimates of patients with specific LBP has not yet been assessed. This study examines coding practices for LBP in outpatient care and the use of imaging across specialist groups over a six-year period. Our results are contrasted with the perspectives presented in the aforementioned guidelines to better understand the strengths and limitations of the ICD-10 coding system in measuring the quality of care for LBP.

## Methods

### Study design

We use data from the “Study of Health in Pomerania” (SHIP), a population-based research study conducted in the administrative districts of Vorpommern-Rügen and Vorpommern-Greifswald in Mecklenburg-Western Pomerania in Germany [16]. SHIP investigates a large number of prevalent diseases, their treatment and risk factors [16].

The SHIP study comprises three independent cohorts, SHIP-START, SHIP-TREND and SHIP-NEXT [16, 17]. Each cohort was recruited as a general population sample from Mecklenburg-Western Pomerania, with participants aged 20–79 years [16, 17]. Baseline examinations for SHIP-START took place between 1997 and 2001, for SHIP-TREND between 2008 and 2012, and are currently taking place for SHIP-NEXT (i.e., data for this cohort are not yet available for analysis). For the present analysis with an observation period of 2014–2019, we selected the SHIP-TREND cohort as the data source, as it offered the largest sample size during this time period and was less affected by selection bias compared to the SHIP-START cohort, which had already undergone three examinations at this time [17]. Based on claims data for the SHIP-TREND cohort between 2014 and 2019, we conducted a retrospective analysis of the frequency of diagnostic codes for LBP.

### Study cohort

During recruitment for SHIP-TREND, 10,000 individuals were randomly selected from Mecklenburg-Western Pomerania’s population register, stratified by age, gender, and place of residence [16]. After excluding deceased persons and persons who had moved away, 8,826 persons remained, all of whom were invited to participate. Recruitment efforts comprised up to three written invitations, phone calls and home visits. A total of 4,420 people (including 2,275 women) participated in the initial survey in 2008–2012 (response 50.1%) [17]. All participants gave written informed consent. The study protocol was approved by the Ethics Committee of Greifswald University Hospital.

**Fig. 1:**
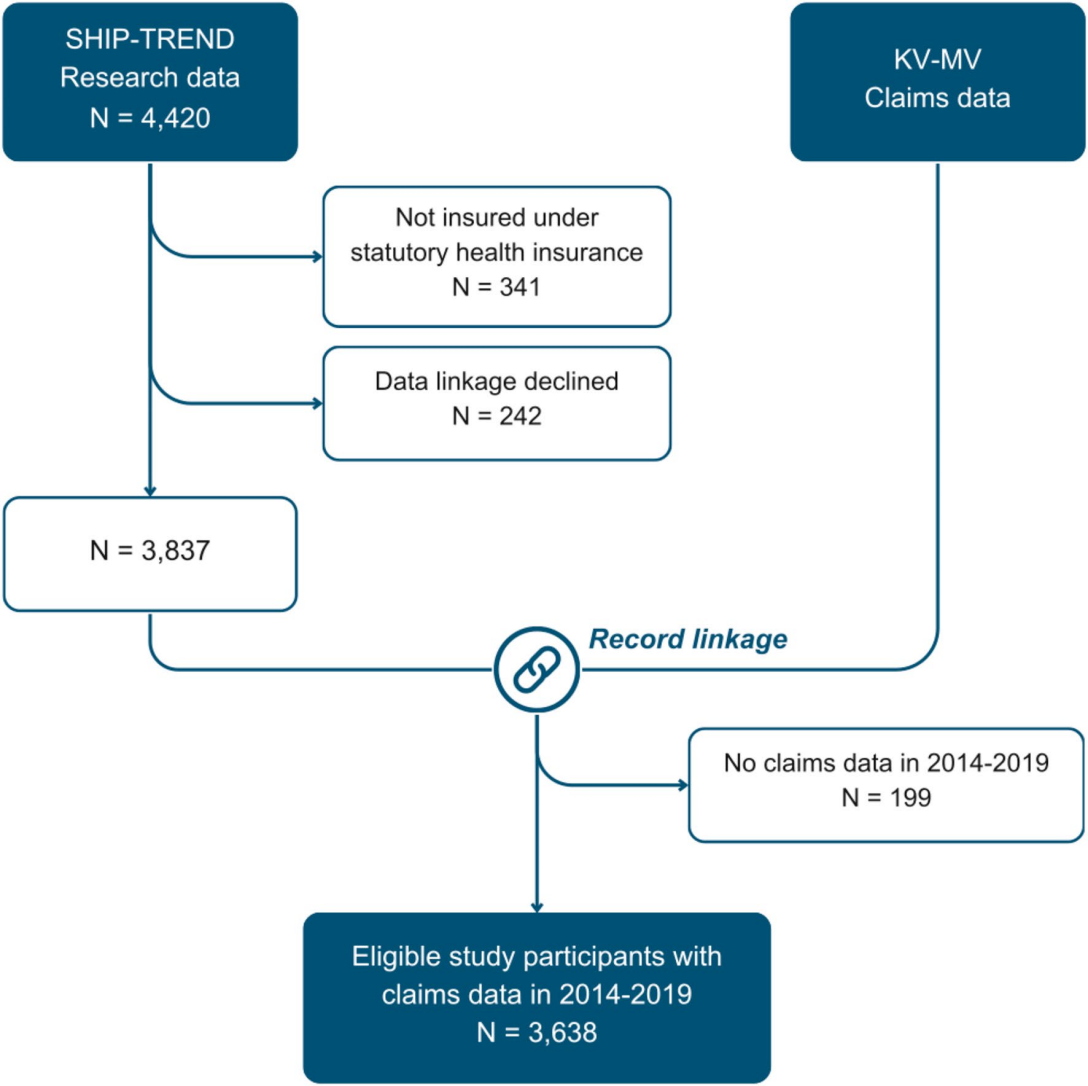
Study design flowchart: cohort selection and data linkage

In addition to research data from SHIP, we use claims data provided by the Mecklenburg-Western Pomeranian Association of Statutory Health Insurance Physicians (Kassenärztliche Vereinigung Mecklenburg-Vorpommern, KV-MV). Of the 4,420 participants in the SHIP-TREND cohort, 4,079 (92.3%) were insured under statutory health insurance. A total of 3,837 study participants (86.8%) with statutory health insurance consented to the use of their claims data (Fig. 1). We linked research data with claims data using participants’ surname, name, date of birth, and sex. The record linkage procedure has been described in detail elsewhere [18]. Claims data were successfully linked for 3,638 participants (82.3%) for the observation period from 2014 to 2019.

### Variables

The claims data include ICD-10 diagnoses as well as fee schedule items. The following ICD categories were considered to be LBP-related: M40 (kyphosis and lordosis), M41 (scoliosis), M42 (spinal osteochondrosis), M43 (other deforming dorsopathies), M45 (ankylosing spondylitis), M46 (other inflammatory spondylopathies), M47 (spondylosis), M48 (other spondylopathies), M51 (other intervertebral disc disorders), M53 (other dorsopathies, not elsewhere classified), and M54 (dorsalgia). Codes for thoracic and cervical localisations were excluded (Table Z2), as well as diagnoses that were only available as three-digit codes, as it cannot be clearly determined at this coding level whether the diagnoses referred to LBP or chest, shoulder or neck pain (2,068 out of 48,351 diagnoses). ICD-10 codes up to the fifth digit were used to quantify diagnoses of specific LBP and to investigate the localisation information. For the latter, we used the following classification:

- unspecified localisation or no localisation: five-digit ICD-10 codes with “9” in the last position or four-digit ICD-10 codes that do not inform on the localisation,
- multiple localisations: five-digit ICD-10 codes with “0” in the last position,
- localisation specified: five-digit ICD-10 codes with “5” to “8” in the last position or four-digit ICD-10 codes referring to a specific localisation (“5” thoracolumbar region, “6” lumbar region, “7” lumbosacral region, “8” sacral and sacrococcygeal region).

Based on the claims data, we inferred which specialist groups were consulted in each quarter, including general practitioners (GPs), orthopaedists, and neurologists or neurosurgeons. The billed fee schedule items indicated whether imaging procedures were used, including X-ray, MRI, and CT (Table Z3).

Data from SHIP were used to describe the baseline characteristics of the participants, including age, sex, (current) smoking status, BMI, physical and psychological quality of life (SF-12 [19]). Detailed information on data assessment can be found elsewhere [16].

### Statistical analyses

We performed descriptive statistical analyses using the statistical software R with the packages tidyverse, waffle and ComplexUpset [20–22]. To describe coding practices for LBP, our analysis included assessing the frequency of ICD-10 codes for LBP diagnoses over time, characterizing the co-occurrence of LBP diagnoses, and investigating the frequency of ICD-10 four- and five-digit codes used to specify LBP localisation. ICD-10 codes and their combinations were assessed per year and during the entire observation period. Within each year, diagnoses may have been coded in different quarters. Thus, the combinations reflect the co-occurrence within the specified time range, without indicating a simultaneous diagnosis. In order to compare coding practices for LBP and the use of diagnostic imaging across specialist groups, we stratified patients with LBP into two groups: those who consulted orthopaedists, neurologists or neurosurgeons during the observation period, and those who were treated solely by GPs. For tentatively classifying patients as having specific LBP, we used the ICD-10 codes listed by the German guideline on specific LBP [13] (Table Z1). A patient was classified as having specific LBP if at least one of these codes was recorded within the specified time period, regardless of any other codes.

## Results

### Frequency of ICD-10 codes used for low back pain diagnoses

Out of 3,837 participants, 2,474 (64.5%) received at least one LBP diagnosis during the entire observation period. 1,180 participants (30.8%) received LBP diagnoses from only a single ICD category, while 1,294 participants (33.7%) received diagnoses from multiple LBP-related categories. 333 participants (8.7%) received a LBP diagnosis in every quarter (Figure Z1).

Table 1 shows the baseline characteristics of the participants. The median age at the start of the observation period was 57 years; 53.5% were women. At the SHIP-TREND baseline examination, 26.6% were smokers, and the median BMI was 27.6 kg/m². Participants for whom claims data were not available were younger and more likely to be male.

**Table 1:** Baseline characteristics

	Participants with statutory health insurance with consent to data linkage (*N* = 3,837)		Participants with statutory health insurance without consent to data linkage (*N* = 242)
	Claims data available (*N* = 3,638)	Claims data not available (*N* = 199)	
Age in years at the beginning of the observation period (2014) (median, interquartile range)	57 (44–69)	50 (32–70)	56 (42–65)
Proportion of women (absolute, relative)	1,946 (53.5%)	76 (38.2%)	131 (54.1%)
Proportion of smokers (absolute, relative; missing data)	968 (26.6%); (18)	61 (30.7%); (3)	57 (23.6%); (1)
BMI in kg/m² (median, interquartile range; missing data)	27.6 (24.6–31.1); (7)	26.7 (23.9–30.9); (0)	26.5 (23.5–30.4); (0)
Sum score SF-12 on physical quality of life (median, interquartile range; missing data)	50 (42–54); (89)	51 (41–55); (8)	50 (42–54); (9)
Sum score SF-12 on psychological quality of life (median, interquartile range; missing data)	55 (49–58); (89)	54 (47–58); (8)	55 (46–58); (9)

The reported characteristics were taken from the SHIP-TREND baseline examination. No claims data were available for 199 participants despite their consent to data linkage. For comparison, the participants with statutory health insurance who refused data linkage are also described. They were excluded from the analysis

Table 2 shows the frequency of LBP diagnoses per ICD category. During the entire observation period, 2,158 participants (56.2%) received at least one diagnosis of dorsalgia (M54). Other intervertebral disc disorders (M51) were coded in 833 (21.7%) and spondylosis (M47) in 803 participants (20.9%). LBP diagnoses from the categories spinal osteochondrosis (M42), other dorsopathies, not elsewhere classified (M53), other spondylopathies (M48), other deforming dorsopathies (M43), kyphosis and lordosis (M40), ankylosing spondylitis (M45), other inflammatory spondylopathies (M46) and scoliosis (M41) were each coded in less than 400 participants (< 10%).

**Table 2:** Frequency of participants with LBP diagnoses by ICD category and year, 2014–2019

Year		2014	2015	2016	2017	2018	2019	Over the entire observation period (2014–2019)
Number of participants with at least one LBP diagnosis		1,431 (37.3%)	1,453 (37.9%)	1,470 (38.3%)	1,473 (38.4%)	1,429 (37.2%)	1,423 (37.1%)	2,474 (64.5%)
ICD-10 category	M54	1,012 (26.4%)	1,048 (27.3%)	1,067 (27.8%)	1,071 (27.9%)	1,024 (26.7%)	1,011 (26.3%)	2,158 (56.2%)
	M51	448 (11.7%)	430 (11.2%)	458 (11.9%)	465 (12.1%)	468 (12.2%)	496 (12.9%)	833 (21.7%)
	M47	430 (11.2%)	431 (11.2%)	425 (11.1%)	431 (11.2%)	414 (10.8%)	454 (11.8%)	803 (20.9%)
	M42	166 (4.3%)	175 (4.6%)	173 (4.5%)	167 (4.4%)	168 (4.4%)	177 (4.6%)	370 (9.6%)
	M53	128 (3.3%)	134 (3.5%)	120 (3.1%)	126 (3.3%)	121 (3.2%)	139 (3.6%)	293 (7.6%)
	M48	115 (3.0%)	116 (3.0%)	125 (3.3%)	140 (3.6%)	154 (4.0%)	155 (4.0%)	281 (7.3%)
	M43	82 (2.1%)	97 (2.5%)	88 (2.3%)	93 (2.4%)	97 (2.5%)	97 (2.5%)	182 (4.7%)
	M40	36 (0.9%)	28 (0.7%)	28 (0.7%)	36 (0.9%)	39 (1.0%)	36 (0.9%)	84 (2.2%)
	M45	16 (0.4%)	16 (0.4%)	19 (0.5%)	14 (0.4%)	15 (0.4%)	18 (0.5%)	34 (0.9%)
	M46	8 (0.2%)	6 (0.2%)	8 (0.2%)	8 (0.2%)	13 (0.3%)	9 (0.2%)	22 (0.6%)
	M41	3 (0.1%)	4 (0.1%)	2 (0.1%)	4 (0.1%)	9 (0.2%)	8 (0.2%)	15 (0.4%)
Number of participants with diagnoses linked to specific LBP		1,060 (27.6%)	1,076 (28.0%)	1,089 (28.4%)	1,103 (28.7%)	1,052 (27.4%)	1,054 (27.5%)	2,121 (55.3%)
Proportion of participants with diagnoses linked to specific LBP in reference to the number of participants with any LBP diagnosis in the respective time period		74.1%	74.1%	74.1%	74.9%	73.6%	74.1%	85.7%

ICD-10 categories: M54 (dorsalgia), M51 (other intervertebral disc disorders), M47 (spondylosis), M42 (spinal osteochondrosis), M53 (other dorsopathies, not elsewhere classified), M48 (other spondylopathies), M43 (other deforming dorsopathies), M40 (kyphosis and lordosis), M45 (ankylosing spondylitis), M46 (other inflammatory spondylopathies), M41 (scoliosis)

Percentages refer to the total number of participants who agreed to data linkage (*N* = 3,837), if not stated otherwise. Multiple diagnoses from different categories per participant occurred

Each year, about 38% of the participants received a LBP diagnosis (Table 2, M = 1447, SD = 21.9). The ICD-10 code for dorsalgia (M54) was assigned to 27% of the participants per year (M = 1039, SD = 26.9), and the codes for other intervertebral disc disorders (M51) and for spondylosis (M47) were assigned to 11–13% (M51: M = 461, SD = 22.0; M47: M = 431, SD = 13.1). The other codes were assigned to less than 5% of the participants per calendar year. The frequency of the codes changed only minimally over the observation period.

Each year, 46–51% of the participants with a dorsalgia diagnosis (M54) received no other LBP diagnosis (Figure Z2). The ICD categories intervertebral disc disorders (M51), spondylosis (M47), and other dorsopathies (M53) occurred as the sole LBP diagnosis in 19–36% of participants with LBP diagnoses of these categories annually, while the categories other spondylopathies (M48) and other deforming dorsopathies (M43) were coded as sole LBP diagnoses in less than 16%. For the ICD category spinal osteochondrosis (M42), the proportion of participants with no additional LBP diagnoses from other categories decreased from approximately 27% in 2014 to 13% in 2019.

### Combinations of low back pain diagnoses

**Fig. 2:**
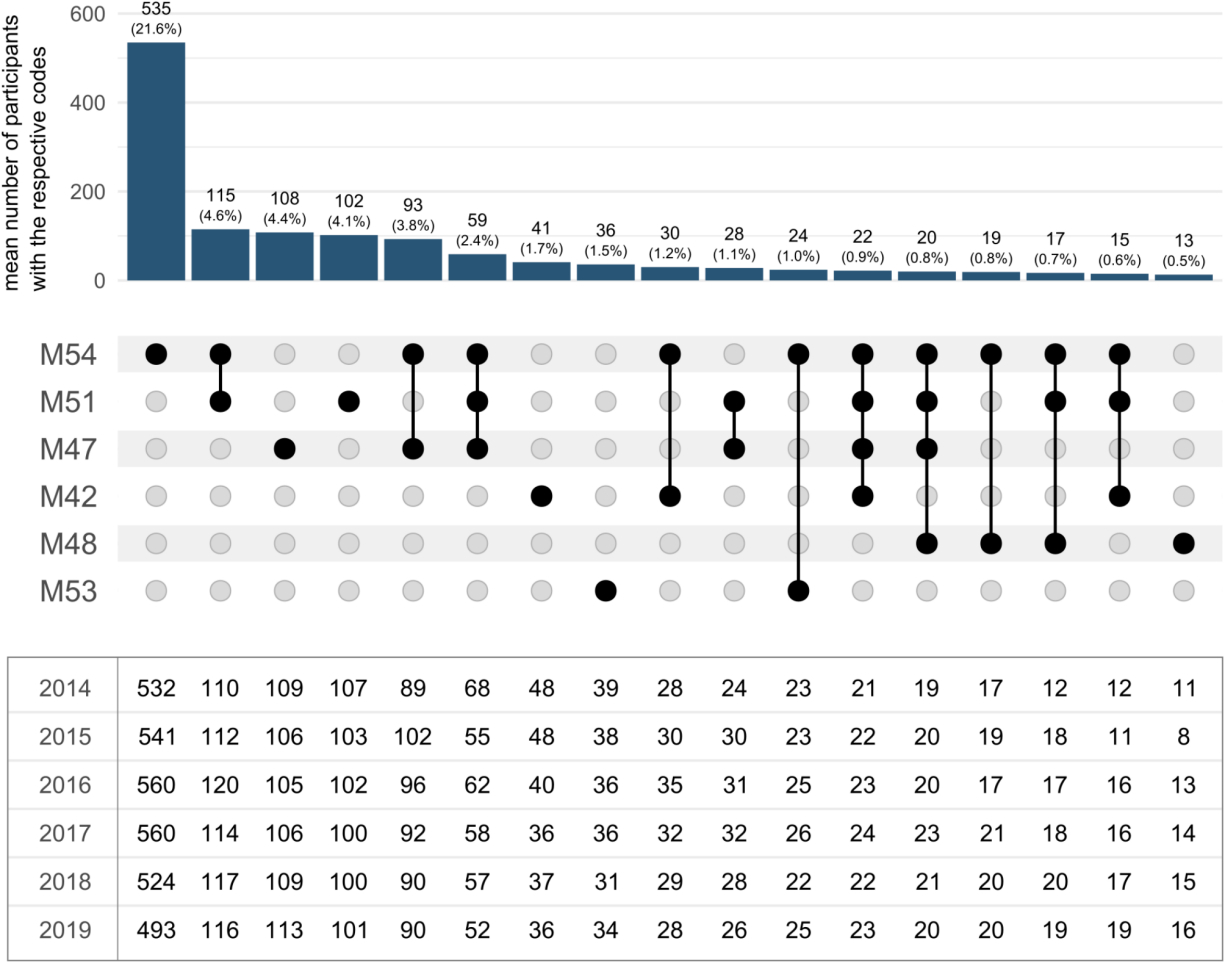
Most frequent LBP diagnoses from single or combined categories within one year. ICD-10 categories: M54 (dorsalgia), M51 (other intervertebral disc disorders), M47 (spondylosis), M42 (spinal osteochondrosis), M48 (other spondylopathies), M53 (other dorsopathies, not elsewhere classified). The combinations of categories are shown in the centre. The table below reports the number of participants per year for each of the combinations. The chart above shows the average annual number of participants relative to the total number of participants with at least one LBP diagnosis during the observation period (*N* = 2,474). The figure only shows combinations that occurred for at least 40 participants during the observation period

Figure 2 shows the most frequent combinations of LBP diagnoses per year. Nearly all combinations involving multiple LBP-related ICD-10 categories included the diagnosis dorsalgia (M54). The most frequent combination was dorsalgia (M54) and other intervertebral disc disorders (M51). On average, this combination occurred in 115 participants per year (4.6% of participants with any LBP diagnosis; SD = 3.6). The combination of dorsalgia (M54) and spondylosis (M47) was the second most common; dorsalgia (M54), other vertebral disc disorders (M51) and spondylosis (M47) was the third most common combination. Combinations involving ICD-10 codes for other deforming dorsopathies (M43), kyphosis and lordosis (M40), ankylosing spondylitis (M45), other inflammatory spondylopathies (M46) or scoliosis (M41) occurred in less than 40 participants over the entire observation period and in less than 15 participants per year. LBP diagnoses from up to seven different ICD categories were coded for individual participants within one year (Table Z4).

### Information on spinal localisation

**Fig. 3:**
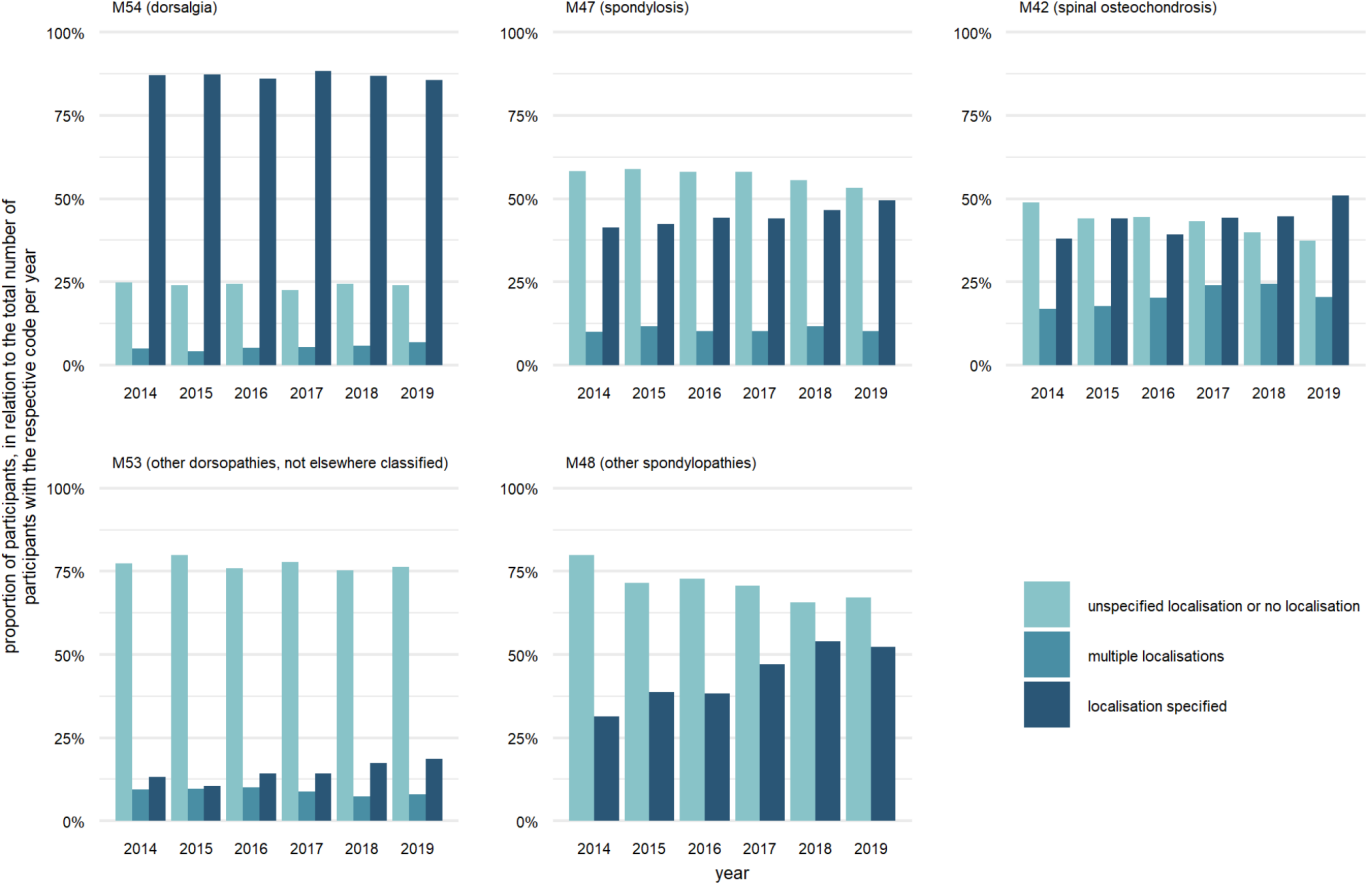
Use of localisation information depicted as proportion of participants per year and ICD-10 category. Percentages refer to the number of participants for whom at least one diagnosis from the specified category was billed in the respective calendar year (see Table 2). Multiple entries of participants per ICD-10 category and year are possible if, for example, both a diagnosis without localisation information and with a specified localisation were billed within one year

Figure 3 shows the proportion of participants with a LBP diagnosis from the categories of dorsalgia (M54), spondylosis (M47), spinal osteochondrosis (M42), other dorsopathies (M53) and other spondylopathies (M48), categorized by localisation. A specific localisation was coded for 87% of participants with dorsalgia (M54) per year. For spondylosis (M47), spinal osteochondrosis (M42) and other spondylopathies (M48), the respective proportion ranged from 31 to 54%. The diagnosis other dorsopathies, not elsewhere classified (M53) was predominantly assigned without localisation information.

Over time, in the categories other spondylopathies (M48), spondylosis (M47) and spinal osteochondrosis (M42), the proportion of participants for whom codes with a specific indication of localisation were billed increased from 31–41% to 50–52% per year. For these ICD categories, the proportion of participants with codes lacking specific localisation information declined (other spondylopathies, M48: from 80% in 2014 to 67% in 2019; spondylosis, M47: from 58 to 53%; spinal osteochondrosis, M42: from 49 to 37%). In category osteochondrosis (M42), the use of codes indicating ‘multiple localisations’ increased from 17% in 2014 to 24% in 2018, before dropping to 20% in 2019.

Frequencies of three-digit and four-digit ICD-10 coded LBP diagnoses are shown in Table Z5. During the entire observation period, 21 different diagnoses were used within the category of dorsalgia (M54). Common diagnoses with a specific spinal localisation were low back pain (M54.5, 33.5% of participants), lumbago with sciatica (M54.4, 25.4% of participants), and ‘radiculopathy: lumbar region’ (M54.16, 15.9% of participants). The most frequent diagnoses without coded localisation were ‘radiculopathy: site unspecified’ (M54.19, 7.5% of participants) and ‘dorsalgia, unspecified: site unspecified’ (M54.99, 8.6% of participants). A total of 15 ICD-10 diagnoses were coded within the category spondylosis (M47), with the most common code being ‘spondylosis, unspecified: site unspecified’ (M47.99), assigned to 7.8% of participants. Eleven different ICD-10 diagnoses were used in the category spinal osteochondrosis (M42). The code for ‘spinal osteochondrosis, unspecified: lumbar region’ (M42.96) was most frequently used. Ten different diagnoses within the category ‘other dorsopathies’ (M53) were used, the most common diagnosis being ‘dorsopathy, unspecified: site unspecified’ (M53.99, 4.3% of participants). A total of six ICD-10 codes were used for other spondylopathies (M48), with the most frequent being ‘spinal stenosis: site unspecified’ (M48.09, 4.7% of all participants) and ‘spinal stenosis: lumbar region’ (M48.06, 3.6% of all participants).

### Coding for specific low back pain

The diagnoses within the category of dorsalgia (M54) linked to specific LBP by the German guideline on specific LBP [13] (i.e. ‘radiculopathy: lumbar region’ (M54.16), lumbago with sciatica (M54.4), lumbago (M54.5), ‘other dorsalgia: lumbosacral region’ (M54.87-8), and ‘dorsalgia, unspecified: lumbosacral region’ (M54.97-8)) occurred in 1,909 participants during the observation period (Table Z5). This leaves 249 participants (11.5%) with diagnoses from the dorsalgia (M54) category not associated with specific LBP. When including all diagnoses linked to specific LBP by the guideline, a total of 2,121 participants received at least one such diagnosis within the observation period (Table 2). If the coded diagnoses are to be interpreted according to the guideline for specific LBP, only 353 participants in our study with a LBP diagnosis would not fall into the category of specific LBP. Therefore, according to the German guideline on specific LBP, the proportion of participants with a diagnosis of specific LBP relative to the total number with LBP in 2014–2019 would be 85.7%. Each year, approximately three out of four participants with a LBP diagnosis received at least one ICD-10 code linked to specific LBP by the guideline (Table 2).

### LBP diagnoses stratified by specialist group

Figure Z3 shows the number of LBP diagnoses per calendar year, stratified by specialist group. Most LBP diagnoses were coded by general practitioners (GPs), with the number increasing from 2014 to 2019. In 2019, 76% of ICD-10 codes were billed by GPs, 16% by orthopaedists, and 7% by neurologists and neurosurgeons.

About half of the participants with a LBP diagnosis were treated exclusively by GPs during the observation period, while the other half were treated at least once or exclusively by another specialist group (Table 3). Among participants who consulted only GPs, 54% had only diagnoses from the dorsalgia (M54) category, while 29% had dorsalgia (M54) along with at least one diagnosis from another LBP-related category during the observation period. Among participants who consulted another specialist group at least once during the observation period, 22% had only dorsalgia (M54) diagnoses, while 69% had dorsalgia (M54) along with at least one diagnosis from another category. When considering diagnoses associated with specific LBP according to the German guideline on specific LBP, 80% of participants who consulted exclusively with their GPs had specific LBP and 92% of those who consulted other specialists had specific LBP. The proportion of participants who received imaging was substantially lower for the group consulting only GPs (22%), as compared to 70% of those who also consulted another specialist group.

**Table 3:** Frequencies of billed ICD-10 categories and imaging by specialist group, 2014–2019

	Only general practitioners	At least one visit to another specialist group (orthopaedists, neurologists or neurosurgeons)
Number of participants	1,154	1,249
Diagnosis of low back pain		
M54 only	626 (54.2%)	272 (21.8%)
M54 and at least one other category	334 (28.9%)	863 (69.1%)
M54 only, excluding diagnoses linked to specific LBP	107 (9.3%)	28 (2.2%)
Diagnoses linked to specific LBP from any ICD-10 category	918 (79.5%)	1,148 (91.9%)
Billing for imaging of the spine (X-ray, MRI or CT)	256 (22.2%)	871 (69.7%)

For 71 participants with a LBP diagnosis, no specialist group could be assigned due to missing fee schedule items.

## Discussion

### Main findings

1. During the six-year observation period, 64% of our general-population sample received at least one diagnosis of LBP. The ICD-10 category dorsalgia (M54) was coded most often. Per calendar year, 38% of all included participants received a LBP diagnosis, and 27% were assigned a diagnosis within the category of dorsalgia (M54). The annual frequencies of these ICD-10 codes changed only minimally during the observation period.
2. 34% of the participants received LBP diagnoses in at least two ICD categories during the observation period. A maximum of seven ICD categories were assigned within the same patient. Around half of the participants with dorsalgia (M54) also had LBP diagnoses from other LBP-related ICD categories within one year, primarily other intervertebral disc disorders (M51) and spondylosis (M47).
3. The ICD-10 codes for specific LBP as specified by the German guideline on specific LBP were assigned at least once in 86% of the participants with a LBP diagnosis during the observation period, far exceeding the expected prevalence.
4. The use of localisation information varied significantly between ICD categories, with a notable percentage of codes for a specific localisation observed particularly in the dorsalgia (M54) category.
5. Around three quarters of LBP cases were coded by GPs, significantly fewer by orthopaedists, neurologists or neurosurgeons. Around half of the participants with LBP diagnoses were treated exclusively by GPs. These participants were much more likely to receive diagnoses only from the category of dorsalgia (M54, 54% vs. 22%) and received imaging procedures less often (22% vs. 70%).

### Comparison to previous studies

The fact that almost two thirds of SHIP-TREND participants received at least one diagnosis of LBP over the course of six years underlines the major epidemiological and health economic significance of LBP. In an analysis of all persons statutorily insured by the “Allgemeine Ortskrankenkasse” in Germany in 2016, around 27% of all insured persons had a diagnosis of LBP based on the ICD-10 category dorsalgia (M54) [23]. In our analysis, the relative frequency of codes for dorsalgia per year was at a similar level (around 29%). However, our analysis also showed that relying solely on the ICD category dorsalgia to identify patients with LBP might lead to an underestimation of the number of patients with LBP per year by approximately 10% points, assuming that the selected ICD codes adequately capture the number of patients with LBP.

The Global Burden of Disease Study analysed data on the prevalence and years lived with disability for LBP from 1990 to 2020 and projected the prevalence rates to 2050 [8]. The analyses were based on three ICD-10 diagnoses within the category of dorsalgia (sciatica (M54.3), lumbago with sciatica (M54.4), low back pain (M54.5)) and the ICD-9 code 724 (low back pain). Our analyses suggest that the prevalence, and thus the projection to 2050, may underestimate the LBP burden, because in each year every second patient with LBP did not receive one of the selected dorsalgia diagnoses, but a different ICD-10 code related to LBP (Table Z6). A consensus on the choice of codes would improve the comparability of health care analyses, which require both transparent reporting of the ICD codes used and a differentiated discussion of the limitations of the data.

Contrary to international estimates, which assume that most LBP is non-specific, our analysis using ICD-10 codes listed in the German guideline on specific LBP indicates that the majority of patients with LBP would have specific underlying causes according to this classification. This finding raises concerns about the link of the ICD-10 codes in this guideline to specific LBP and substantially raises uncertainty about the appropriate use of ICD-10 codes. Consequently, we argue that ICD-10 codes in claims data should not be considered useful tools for measuring process quality of care for LBP.

There is no internationally agreed upon set of indicators to measure the quality of care for LBP [9]. A number of quality indicators have been proposed geared towards non-specific LBP. For example, the 2017 NICE clinical standards for primary care management of LBP proposed indicators that included documentation of risk stratification, lumbar spine imaging in the absence of suspected serious underlying pathology, and documentation of the provision of advice and patient information to promote self-management of symptoms [24]. A recent scoping review identified available structure, process and outcome indicators in grey and published literature [25]. The majority of reported quality indicators are process indicators, and of these, the most common are related to imaging (e.g., total number of X-rays requested, proportion of X-rays for those with red flags), referrals to other healthcare providers, and documentation of shared decision-making.

### Strengths and limitations

Our analyses are based on a large population-based sample of the predominantly rural area of Mecklenburg-Western Pomerania in Germany. The data cover a six-year long billing period. However, it should be noted that there are some limitations to the study.

Mecklenburg-Western Pomerania is the most sparsely populated federal state in Germany. The population is on average older, has seen a substantial population decline over time and faces higher unemployment rates compared to the German national average. Most persons in Mecklenburg-Western Pomerania live in rural areas, which affects access to healthcare. In our sample, only few consulted another specialist without GP contact. Therefore, our results may not be generalizable to urban areas, where there is a higher density of and easier direct access to specialists other than GPs. In urban areas, we would expect a higher percentage of spinal imaging and more different diagnoses during a given billing period [26].

ICD-10 codes are primarily used for billing purposes [27] and may not adequately reflect morbidity, as the ICD-10 system does not cover all entities of LBP [28]. Moreover, chronic diseases, such as chronic LBP, may be recorded even when not being treated at the time [29]. In addition, the coding practice of the treating physician may be subject to the influence of individual and regional coding habits as well as economic factors [30]. There is no established documentation standard for the history of present illness, the results of the physical examination and the contents of the counselling, which are an important part of the guideline recommendations [31].

Claims data were not available for privately insured persons. This group generally consists of younger, healthier, and more socially advantaged individuals. However, health service reports are also primarily based on claims data from statutorily insured individuals, and the proportion of this particular group is low (approx. 6% of the cohort).

Pain in the lumbosacral region is the most common [32]. Therefore, we included the ICD-10 codes for back pain in multiple localisations or without a specified localisation in our analysis. This may have led to a slight overestimation of cases with LBP.

### Implications of the findings

The uncertainty in inferring the underlying cause from an ICD-10 code impacts assessing the quality of care for patients with LBP. Since categorisation into specific and non-specific LBP seems mostly uncertain, it would be more pragmatic to formulate a target range for the provision of various care services for patients with LBP. This could be, for example, a defined proportion of patients with LBP who receive imaging, opioids, or invasive therapies. These target ranges could be based on comparisons with regional or international care data.

## Conclusion

Our analysis underscores the high epidemiological relevance of LBP, as almost two thirds of the participants received a diagnosis over a six-year period. When applying the German guideline on specific LBP to link ICD-10 codes with an assumed specific origin, most LBP would be classified as specific—contrary to common international assumptions. This suggests that the guideline’s approach to linking diagnostic codes with specific LBP is not suited for identifying patients with specific LBP using claims data. It also highlights considerable problems in using ICD-10 codes to assess quality of care for LBP. The lack of a uniform reporting standard for LBP in health care analyses, combined with the limitations of claims data, underscores the need for transparent reporting of ICD-10 codes used and open discussion of data limitations in health services research.

## Electronic supplementary material

Below is the link to the electronic supplementary material.

Supplementary Material 1

## Data Availability

The claims data used in this study are not publicly available due to privacy restrictions. Data of the SHIP studies are available and can be applied for under https://transfer.ship-med.uni-greifswald.de/FAIRequest/.

## Abbreviations

BMI: Body mass index
CT: Computed tomography
GP: General practitioner
ICD: International Statistical Classification of Diseases and Related Health Problems
KV-MV: Mecklenburg-Western Pomeranian Association of Statutory Health Insurance Physicians (‘Kassenärztliche Vereinigung Mecklenburg-Vorpommern’)
LBP: Low back pain
MRI: Magnetic resonance imaging
QISA: Quality indicator system for outpatient care (‘Qualitätsindikatorensystem für die ambulante Versorgung’)
SF-12: 12-item short form health survey
SHIP: Study of Health in Pomerania
