# Supplementary material for: Frequency, combinations and clinical relevance of outpatient low back pain diagnoses: analysis of claims data in a population-based cohort study
Source: BMC Musculoskelet Disord. 2025 Apr 3;26:330. doi: 10.1186/s12891-025-08514-1 (PMC11966858; doi:10.1186/s12891-025-08514-1)
Supplement: Supplementary file 1 — Supplementary Material 1 [file 12891_2025_8514_MOESM1_ESM.docx]

# Supplementary material

**Table Z1**: Overview of diagnoses for specific low back pain (LBP) based on the German guideline on specific LBP [1].

| ICD-10 category | Description | ICD-10 codes named in the guideline | Prevalence assumption in the guideline |
| --- | --- | --- | --- |
| *Morphological entities* | | | |
| M47,  M54 | Lumbar facet syndrome / spondylarthrosis | M47.26, M47.27, M47.29, M47.86-88, M47.99, M54.5 | 10-41% of patients with chronic LBP |
| M42,  M47,  M54 | Discogenic lumbar syndrome to vertebral osteochondrosis | M42.16-19, M42.90, M42.96-99, M47.26, M54.16 | 26-39% of patients with chronic LBP without radicular symptoms |
| M45,  M46 | Ankylosing spondylitis | M45.0, M46.8 | 5% of patients with chronic back pain |
| M48 | Baastrup's disease | M48.2 | Age-dependent 40-80%, with bursitis approx. 8% |
| M48,  M99 | Spinal stenosis | M48.06 with M99.33, M48.06 with M99.43, M99.53, M55.3^a^ | Incidence 5 per 100,000 inhabitants per year |
| M43 | Spondylolysis and spondylolisthesis | M43.0, M43.1 | Incidence up to 11.5% |
| M19,  M24,  M54,  M70,  M99 | Pathological processes in the sacroiliac joints | M19.05, M19.25, M19.85, M19.95, M24.25, M54.4, M54.5, M54.87, M54.88, M54.97, M54.98, M70.8, M99.03, M99.04, M99.83, M99.84 | 15-30% of patients with LBP |
| *Functional entities* | | | |
| M62,  M79,  M99 | Myofascial dysfunction (somatic dysfunction, myogelosis, atrophy of inactivity) | M62.59, M62.85, M62.88, M62.9, M62.98, M79.1, M99.0 | Frequent, not assessable |
|  | Hypomobile segmental dysfunction of the lumbar spine (blockage) | No ICD code given | Frequent concomitant pathology |

^a^ This code is not included in ICD-10 and might be a typo (G55.3?).

[1] Deutsche Gesellschaft für Orthopädie und Unfallchirurgie e.V. Spezifischer Kreuzschmerz: Version 2.0 2024. (available at *https://register.awmf.org/de/leitlinien/detail/187‐059*)

**Table Z2**: Overview of excluded ICD-10 subcategories.

| ICD-10 category | Description | Excluded subcategories |
| --- | --- | --- |
| M40 | Kyphosis and lordosis | M40.01-4, M40.11-4, M40.21-4, M40.31-4, M40.41-4, M40.51-4 |
| M41 | Scoliosis | M41.01-4, M41.11-4, M41.21-4, M41.3, M41.3x, M41.4, M41.4x, M41.5, M41.5x, M41.8, M41.8x, M41.9, M41.9x |
| M42 | Spinal osteochondrosis | M42.01-4, M42.11-4, M42.91-4 |
| M43 | Other deforming dorsopathies | M43.01-4, M43.11-4, M43.21-4, M43.3, M43.4, M43.5, M43.5x, M43.6, M43.8, M43.80-4, M43.91-4 |
| M45 | Ankylosing spondylitis | M45.01-4 |
| M46 | Other inflammatory spondylopathies | M46.01-4, M46.21-4, M46.31-4, M46.41-4, M46.51-4, M46.81-4, M46.91-4 |
| M47 | Spondylosis | M47.01-4, M47.11-4, M47.21-4, M47.81-4, M47.91-4 |
| M48 | Other spondylopathies | M48.01-4, M48.11-4, M48.21-4, M48.31-4, M48.41-4, M48.51-4, M48.81-4, M48.91-4 |
| M51 | Other intervertebral disc disorders |  |
| M53 | Other dorsopathies, not elsewhere classified | M53.0, M53.1, M53.21-4, M53.81-4, M53.91-4 |
| M54 | Dorsalgia | M54.01-4, M54.11-4, M54.2, M54.6, M54.81-4, M54.91-4 |

Codes for thoracic and cervical localisations were excluded, but not diagnoses without localisation information or with the specification of multiple localisations, assuming that most patients have pain in the lumbosacral region [2].

[2] Sendera M, Sendera A. Rückenschmerz. In: Sendera M, Sendera A, editors. Chronischer Schmerz, Vienna: Springer; 2015, p. 79–85. https://doi.org/10.1007/978-3-7091-1841-2_8.

**Table Z3**: Overview of the included fee schedule items.

| Description | Fee schedule items |
| --- | --- |
| General practitioners | 03003, 03004, 03005, 03220, 03111, 03112 |
| Orthopaedists | 18211, 18212, 18331 |
| Neurologists or neurosurgeons | 16211, 16212, 16232 |
| Imaging (X-ray, MRI, CT) | 34221, 34222, 34223, 34311, 34411 |


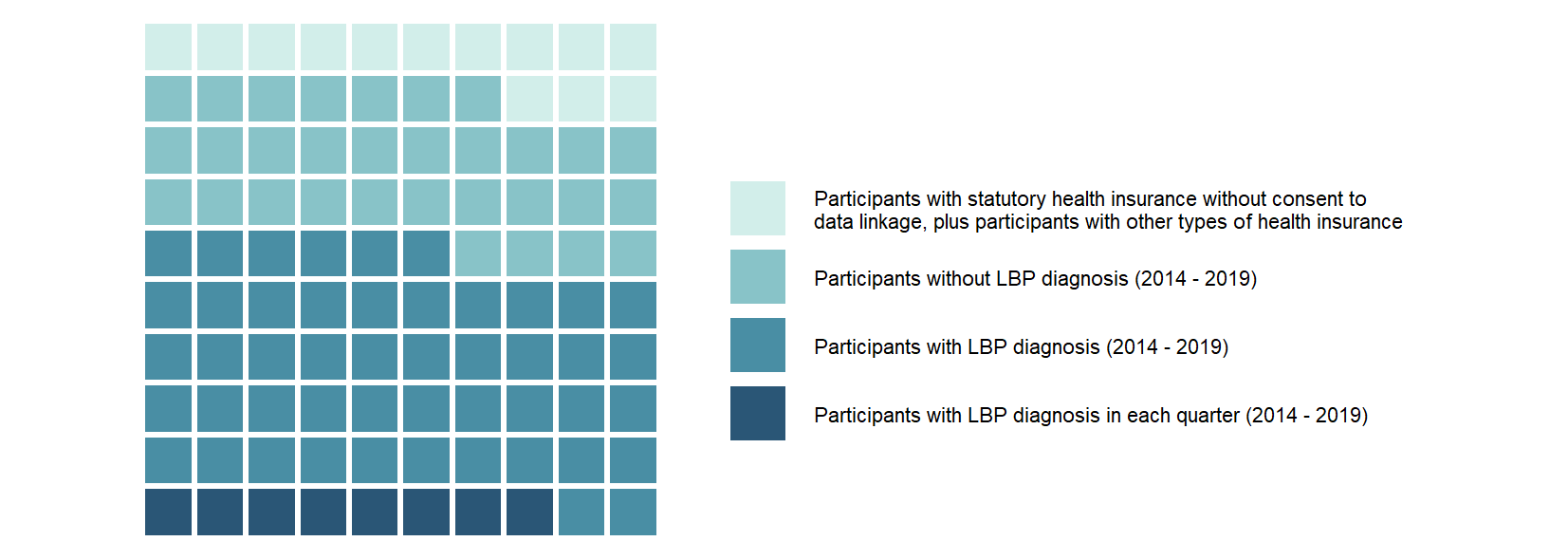


**Figure Z1:** Availability of billing data for the SHIP-TREND participants (N = 4,420) and proportion of participants for whom LBP diagnoses were coded in 2014–2019. One box corresponds to approximately 44 persons.


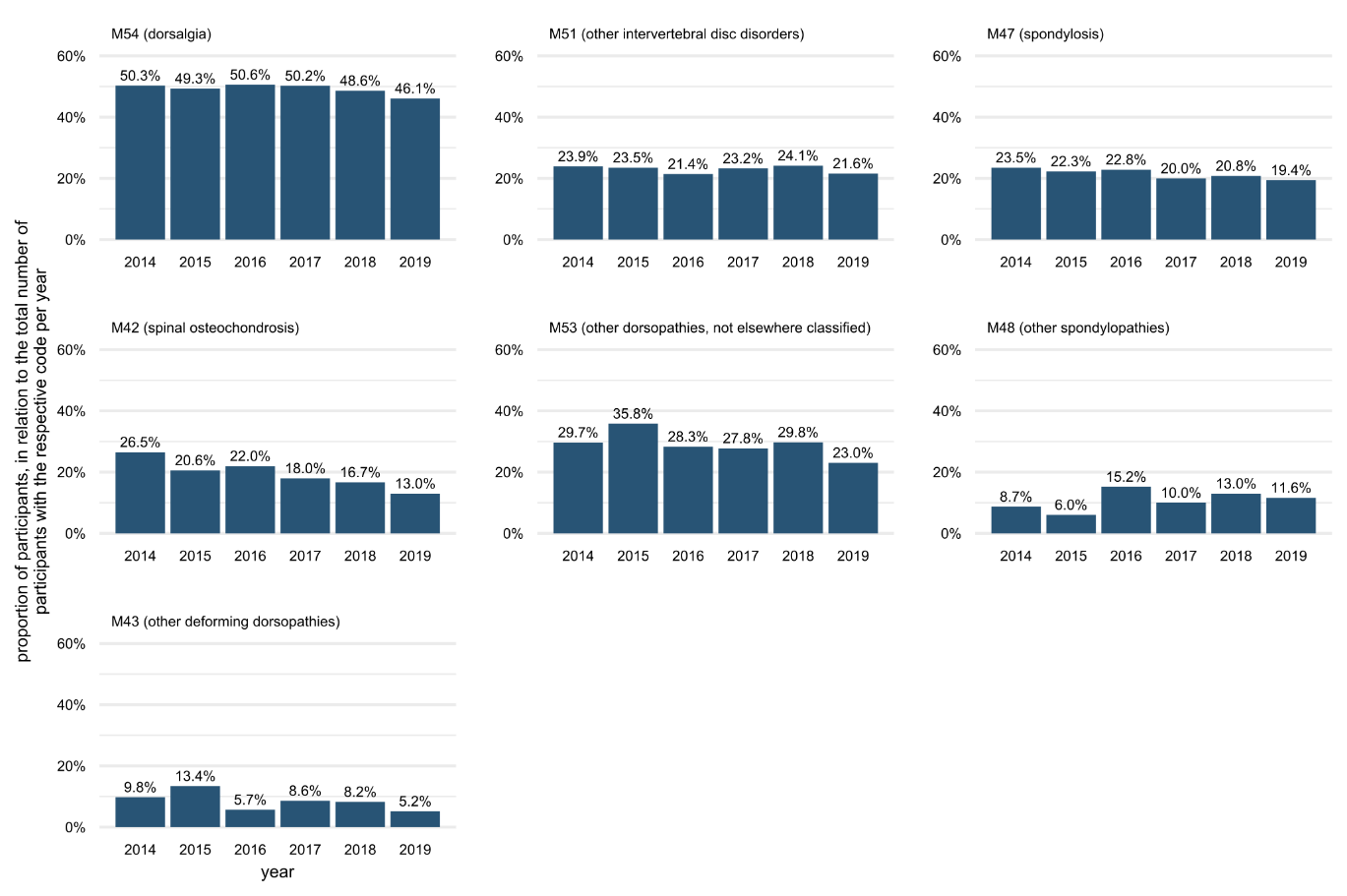


**Figure Z2:** Proportion of participants who received a LBP diagnosis from a single ICD-10 category. Percentages refer to the total number of participants for whom at least one diagnosis from the specified category was billed in the respective year (see Table 2). The categories M40, M45, M46 and M41 are not shown due to their low case numbers.

**Table Z4:** Number of participants who received diagnoses from only one or up to seven different ICD categories within one year or within the entire observation period. Percentages refer to the number of participants with statutory health insurance and consent to data linkage (N = 3,837).

| ****Year**** | ****2014**** | ****2015**** | ****2016**** | ****2017**** | ****2018**** | ****2019**** | ****Over the entire observation period (2014-2019)**** |
| --- | --- | --- | --- | --- | --- | --- | --- |
| Diagnoses from one category | 828 (21.6%) | 829 (21.6%) | 842 (21.9%) | 835 (21.8%) | 806 (21.0%) | 758 (19.8%) | 1180 (30.8%) |
| Diagnoses from two categories | 344 (9.0%) | 357 (9.3%) | 344 (9.0%) | 352 (9.2%) | 342 (8.9%) | 359 (9.4%) | 577 (15.0%) |
| Diagnoses from three categories | 153 (4.0%) | 165 (4.3%) | 192 (5.0%) | 180 (4.7%) | 158 (4.1%) | 177 (4.6%) | 351 (9.1%) |
| Diagnoses from four categories | 69 (1.8%) | 71 (1.9%) | 63 (1.6%) | 63 (1.6%) | 72 (1.9%) | 72 (1.9%) | 214 (5.6%) |
| Diagnoses from five categories | 29 (0.8%) | 24 (0.6%) | 20 (0.5%) | 36 (0.9%) | 37 (1.0%) | 39 (1.0%) | 96 (2.5%) |
| Diagnoses from six categories | 8 (0.2%) | 6 (0.2%) | 8 (0.2%) | 5 (0.1%) | 13 (0.3%) | 16 (0.4%) | 40 (1.0%) |
| Diagnoses from seven categories | 0 (0.0%) | 1 (0.0%) | 1 (0.0%) | 2 (0.1%) | 1 (0.0%) | 2 (0.1%) | 16 (0.4%) |

**Table Z5:** Absolute and relative frequencies of participants with billed LBP diagnoses per year and for the entire observation period. Percentages refer to the number of participants with statutory health insurance and consent to data linkage (N = 3,837).

| Cat. | ICD Code | 2014 | 2015 | 2016 | 2017 | 2018 | 2019 | ****Over the entire period (2014-2019)**** |
| --- | --- | --- | --- | --- | --- | --- | --- | --- |
| M54 | M54.1 | 18 (0.5%) | 18 (0.5%) | 20 (0.5%) | 17 (0.4%) | 11 (0.3%) | 11 (0.3%) | 36 (0.9%) |
|  | M54.10 | 27 (0.7%) | 22 (0.6%) | 33 (0.9%) | 31 (0.8%) | 32 (0.8%) | 44 (1.1%) | 98 (2.6%) |
|  | M54.15 | 10 (0.3%) | 12 (0.3%) | 18 (0.5%) | 23 (0.6%) | 18 (0.5%) | 24 (0.6%) | 76 (2.0%) |
|  | M54.16 | 220 (5.7%) | 221 (5.8%) | 227 (5.9%) | 238 (6.2%) | 248 (6.5%) | 243 (6.3%) | 611 (15.9%) |
|  | M54.17 | 50  (1.3%) | 56  (1.5%) | 70  (1.8%) | 75  (2.0%) | 73  (1.9%) | 79  (2.1%) | 219 (5.7%) |
|  | M54.18 | 5 (0.1%) | 4 (0.1%) | 3 (0.1%) | 4 (0.1%) | 9 (0.2%) | 8 (0.2%) | 14 (0.4%) |
|  | M54.19 | 122 (3.2%) | 126 (3.3%) | 123 (3.2%) | 115 (3.0%) | 118 (3.1%) | 117 (3.0%) | 287 (7.5%) |
|  | M54.3 | 24  (0.6%) | 26  (0.7%) | 19  (0.5%) | 19  (0.5%) | 22  (0.6%) | 19  (0.5%) | 104 (2.7%) |
|  | M54.4 | 359 (9.4%) | 347 (9.0%) | 356 (9.3%) | 385 (10.0%) | 379 (9.9%) | 353 (9.2%) | 976 (25.4%) |
|  | M54.5 | 426 (11.1%) | 483 (12.6%) | 487 (12.7%) | 466 (12.1%) | 420 (10.9%) | 430 (11.2%) | 1.285 (33.5%) |
|  | M54.8 | 1 (0.0%) | 0 (0.0%) | 0 (0.0%) | 0 (0.0%) | 2 (0.1%) | 0 (0.0%) | 3 (0.1%) |
|  | M54.80 | 2 (0.1%) | 3 (0.1%) | 7 (0.2%) | 7 (0.2%) | 9 (0.2%) | 6 (0.2%) | 15 (0.4%) |
|  | M54.86 | 13 (0.3%) | 7 (0.2%) | 10 (0.3%) | 6 (0.2%) | 8 (0.2%) | 13 (0.3%) | 27 (0.7%) |
|  | M54.87 | 0 (0.0%) | 0 (0.0%) | 1 (0.0%) | 1 (0.0%) | 1 (0.0%) | 3 (0.1%) | 4 (0.1%) |
|  | M54.89 | 34 (0.9%) | 35 (0.9%) | 30 (0.8%) | 31 (0.8%) | 30 (0.8%) | 24 (0.6%) | 73 (1.9%) |
|  | M54.9 | 13 (0.3%) | 8 (0.2%) | 6 (0.2%) | 4 (0.1%) | 7 (0.2%) | 8 (0.2%) | 28 (0.7%) |
|  | M54.90 | 20 (0.5%) | 19 (0.5%) | 14 (0.4%) | 19 (0.5%) | 18 (0.5%) | 18 (0.5%) | 42 (1.1%) |
|  | M54.95 | 2 (0.1%) | 2 (0.1%) | 2 (0.1%) | 3 (0.1%) | 4 (0.1%) | 7 (0.2%) | 8 (0.2%) |
|  | M54.96 | 20 (0.5%) | 28 (0.7%) | 27 (0.7%) | 23 (0.6%) | 16 (0.4%) | 23 (0.6%) | 75 (2.0%) |
|  | M54.97 | 27 (0.7%) | 23 (0.6%) | 21 (0.5%) | 34 (0.9%) | 24 (0.6%) | 29 (0.8%) | 95 (2.5%) |
|  | M54.99 | 83  (2.2%) | 88  (2.3%) | 97  (2.5%) | 91  (2.4%) | 90  (2.3%) | 95  (2.5%) | 329 (8.6%) |
| M51 | M51.0 | 7 (0.2%) | 9 (0.2%) | 8 (0.2%) | 8 (0.2%) | 6 (0.2%) | 7 (0.2%) | 11 (0.3%) |
|  | M51.1 | 104 (2.7%) | 99  (2.6%) | 106 (2.8%) | 108 (2.8%) | 109 (2.8%) | 119 (3.1%) | 271 (7.1%) |
|  | M51.2 | 259 (6.8%) | 258 (6.7%) | 284 (7.4%) | 280 (7.3%) | 290 (7.6%) | 311 (8.1%) | 566 (14.8%) |
|  | M51.3 | 40 (1.0%) | 35 (0.9%) | 42 (1.1%) | 45 (1.2%) | 41 (1.1%) | 42 (1.1%) | 88 (2.3%) |
|  | M51.8 | 9 (0.2%) | 11 (0.3%) | 9 (0.2%) | 4 (0.1%) | 6 (0.2%) | 10 (0.3%) | 34 (0.9%) |
|  | M51.9 | 153 (4.0%) | 149 (3.9%) | 156 (4.1%) | 158 (4.1%) | 173 (4.5%) | 167 (4.4%) | 326 (8.5%) |
| M47 | M47.2 | 9 (0.2%) | 8 (0.2%) | 7 (0.2%) | 7 (0.2%) | 5 (0.1%) | 5 (0.1%) | 11 (0.3%) |
|  | M47.20 | 10 (0.3%) | 12 (0.3%) | 9 (0.2%) | 11 (0.3%) | 9 (0.2%) | 10 (0.3%) | 23 (0.6%) |
|  | M47.26 | 90  (2.3%) | 79  (2.1%) | 78  (2.0%) | 71  (1.9%) | 65  (1.7%) | 79  (2.1%) | 216 (5.6%) |
|  | M47.27 | 11 (0.3%) | 14 (0.4%) | 16 (0.4%) | 11 (0.3%) | 17 (0.4%) | 15 (0.4%) | 35 (0.9%) |
|  | M47.29 | 45  (1.2%) | 44  (1.1%) | 43  (1.1%) | 51  (1.3%) | 46  (1.2%) | 50  (1.3%) | 123 (3.2%) |
|  | M47.8 | 10 (0.3%) | 12 (0.3%) | 6 (0.2%) | 6 (0.2%) | 4 (0.1%) | 4 (0.1%) | 13 (0.3%) |
|  | M47.80 | 4 (0.1%) | 7 (0.2%) | 4 (0.1%) | 5 (0.1%) | 6 (0.2%) | 5 (0.1%) | 9 (0.2%) |
|  | M47.86 | 68  (1.8%) | 81  (2.1%) | 78  (2.0%) | 88  (2.3%) | 96  (2.5%) | 115 (3.0%) | 199 (5.2%) |
|  | M47.87 | 9 (0.2%) | 10 (0.3%) | 11 (0.3%) | 17 (0.4%) | 16 (0.4%) | 21 (0.5%) | 41 (1.1%) |
|  | M47.89 | 31 (0.8%) | 31 (0.8%) | 29 (0.8%) | 25 (0.7%) | 20 (0.5%) | 27 (0.7%) | 70 (1.8%) |
|  | M47.9 | 12 (0.3%) | 13 (0.3%) | 7 (0.2%) | 7 (0.2%) | 5 (0.1%) | 5 (0.1%) | 20 (0.5%) |
|  | M47.90 | 29 (0.8%) | 32 (0.8%) | 30 (0.8%) | 28 (0.7%) | 34 (0.9%) | 32 (0.8%) | 50 (1.3%) |
|  | M47.96 | 24 (0.6%) | 17 (0.4%) | 23 (0.6%) | 24 (0.6%) | 20 (0.5%) | 22 (0.6%) | 48 (1.3%) |
|  | M47.97 | 0 (0.0%) | 2 (0.1%) | 3 (0.1%) | 5 (0.1%) | 5 (0.1%) | 4 (0.1%) | 6 (0.2%) |
|  | M47.99 | 170 (4.4%) | 171 (4.5%) | 173 (4.5%) | 174 (4.5%) | 168 (4.4%) | 172 (4.5%) | 298 (7.8%) |
| M42 | M42.09 | 12 (0.3%) | 11 (0.3%) | 13 (0.3%) | 10 (0.3%) | 10 (0.3%) | 13 (0.3%) | 22 (0.6%) |
|  | M42.1 | 17 (0.4%) | 14 (0.4%) | 14 (0.4%) | 12 (0.3%) | 10 (0.3%) | 9 (0.2%) | 19 (0.5%) |
|  | M42.10 | 28 (0.7%) | 31 (0.8%) | 35 (0.9%) | 40 (1.0%) | 41 (1.1%) | 36 (0.9%) | 67 (1.7%) |
|  | M42.16 | 16 (0.4%) | 17 (0.4%) | 16 (0.4%) | 23 (0.6%) | 30 (0.8%) | 35 (0.9%) | 65 (1.7%) |
|  | M42.17 | 8 (0.2%) | 9 (0.2%) | 10 (0.3%) | 11 (0.3%) | 13 (0.3%) | 12 (0.3%) | 21 (0.5%) |
|  | M42.19 | 6 (0.2%) | 7 (0.2%) | 6 (0.2%) | 10 (0.3%) | 5 (0.1%) | 4 (0.1%) | 21 (0.5%) |
|  | M42.9 | 6 (0.2%) | 7 (0.2%) | 7 (0.2%) | 7 (0.2%) | 9 (0.2%) | 7 (0.2%) | 10 (0.3%) |
|  | M42.90 | 8 (0.2%) | 6 (0.2%) | 8 (0.2%) | 6 (0.2%) | 8 (0.2%) | 7 (0.2%) | 17 (0.4%) |
|  | M42.96 | 36  (0.9%) | 46  (1.2%) | 34  (0.9%) | 35  (0.9%) | 30  (0.8%) | 43  (1.1%) | 132 (3.4%) |
|  | M42.97 | 6 (0.2%) | 6 (0.2%) | 9 (0.2%) | 8 (0.2%) | 10 (0.3%) | 11 (0.3%) | 18 (0.5%) |
|  | M42.99 | 33 (0.9%) | 34 (0.9%) | 30 (0.8%) | 29 (0.8%) | 27 (0.7%) | 28 (0.7%) | 56 (1.5%) |
| M53 | M53.26 | 4 (0.1%) | 3 (0.1%) | 3 (0.1%) | 6 (0.2%) | 12 (0.3%) | 13 (0.3%) | 19 (0.5%) |
|  | M53.27 | 5 (0.1%) | 4 (0.1%) | 5 (0.1%) | 6 (0.2%) | 4 (0.1%) | 4 (0.1%) | 10 (0.3%) |
|  | M53.3 | 9 (0.2%) | 14 (0.4%) | 9 (0.2%) | 6 (0.2%) | 6 (0.2%) | 12 (0.3%) | 33 (0.9%) |
|  | M53.8 | 1 (0.0%) | 0 (0.0%) | 1 (0.0%) | 1 (0.0%) | 0 (0.0%) | 1 (0.0%) | 1 (0.0%) |
|  | M53.80 | 12 (0.3%) | 13 (0.3%) | 12 (0.3%) | 11 (0.3%) | 9 (0.2%) | 11 (0.3%) | 28 (0.7%) |
|  | M53.86 | 7 (0.2%) | 6 (0.2%) | 7 (0.2%) | 6 (0.2%) | 5 (0.1%) | 9 (0.2%) | 13 (0.3%) |
|  | M53.89 | 22 (0.6%) | 23 (0.6%) | 18 (0.5%) | 19 (0.5%) | 21 (0.5%) | 20 (0.5%) | 34 (0.9%) |
|  | M53.9 | 2 (0.1%) | 4 (0.1%) | 2 (0.1%) | 1 (0.0%) | 2 (0.1%) | 1 (0.0%) | 4 (0.1%) |
|  | M53.96 | 1 (0.0%) | 1 (0.0%) | 2 (0.1%) | 0 (0.0%) | 0 (0.0%) | 0 (0.0%) | 3 (0.1%) |
|  | M53.99 | 67  (1.7%) | 68  (1.8%) | 63  (1.6%) | 72  (1.9%) | 63  (1.6%) | 73  (1.9%) | 165 (4.3%) |
| M48 | M48.0 | 5 (0.1%) | 6 (0.2%) | 2 (0.1%) | 2 (0.1%) | 3 (0.1%) | 4 (0.1%) | 8 (0.2%) |
|  | M48.06 | 34  (0.9%) | 43  (1.1%) | 42  (1.1%) | 55  (1.4%) | 80  (2.1%) | 78  (2.0%) | 137 (3.6%) |
|  | M48.09 | 78  (2.0%) | 69  (1.8%) | 81  (2.1%) | 89  (2.3%) | 92  (2.4%) | 93  (2.4%) | 181 (4.7%) |
|  | M48.55 | 4 (0.1%) | 3 (0.1%) | 4 (0.1%) | 7 (0.2%) | 6 (0.2%) | 5 (0.1%) | 9 (0.2%) |
|  | M48.56 | 2 (0.1%) | 4 (0.1%) | 4 (0.1%) | 6 (0.2%) | 5 (0.1%) | 6 (0.2%) | 13 (0.3%) |
|  | M48.59 | 9 (0.2%) | 8 (0.2%) | 8 (0.2%) | 9 (0.2%) | 7 (0.2%) | 8 (0.2%) | 15 (0.4%) |
| M43 | M43.09 | 3 (0.1%) | 6 (0.2%) | 5 (0.1%) | 5 (0.1%) | 4 (0.1%) | 3 (0.1%) | 9 (0.2%) |
|  | M43.1 | 7 (0.2%) | 6 (0.2%) | 7 (0.2%) | 9 (0.2%) | 8 (0.2%) | 6 (0.2%) | 13 (0.3%) |
|  | M43.16 | 25 (0.7%) | 27 (0.7%) | 33 (0.9%) | 37 (1.0%) | 39 (1.0%) | 37 (1.0%) | 70 (1.8%) |
|  | M43.17 | 11 (0.3%) | 11 (0.3%) | 9 (0.2%) | 8 (0.2%) | 11 (0.3%) | 13 (0.3%) | 25 (0.7%) |
|  | M43.19 | 36 (0.9%) | 36 (0.9%) | 35 (0.9%) | 37 (1.0%) | 40 (1.0%) | 35 (0.9%) | 78 (2.0%) |
|  | M43.2 | 1 (0.0%) | 1 (0.0%) | 1 (0.0%) | 0 (0.0%) | 0 (0.0%) | 0 (0.0%) | 1 (0.0%) |
|  | M43.29 | 12 (0.3%) | 16 (0.4%) | 12 (0.3%) | 13 (0.3%) | 12 (0.3%) | 13 (0.3%) | 32 (0.8%) |
| M40 | M40.0 | 2 (0.1%) | 2 (0.1%) | 2 (0.1%) | 5 (0.1%) | 3 (0.1%) | 2 (0.1%) | 5 (0.1%) |
|  | M40.2 | 1 (0.0%) | 0 (0.0%) | 0 (0.0%) | 1 (0.0%) | 0 (0.0%) | 0 (0.0%) | 2 (0.1%) |
|  | M40.29 | 25 (0.7%) | 21 (0.5%) | 20 (0.5%) | 23 (0.6%) | 29 (0.8%) | 28 (0.7%) | 59 (1.5%) |
|  | M40.46 | 6 (0.2%) | 3 (0.1%) | 4 (0.1%) | 3 (0.1%) | 3 (0.1%) | 1 (0.0%) | 9 (0.2%) |
|  | M40.56 | 3 (0.1%) | 2 (0.1%) | 3 (0.1%) | 5 (0.1%) | 4 (0.1%) | 5 (0.1%) | 12 (0.3%) |
| M45 | M45.09 | 16 (0.4%) | 16 (0.4%) | 19 (0.5%) | 14 (0.4%) | 15 (0.4%) | 18 (0.5%) | 34 (0.9%) |
| M46 | M46.1 | 5 (0.1%) | 3 (0.1%) | 4 (0.1%) | 4 (0.1%) | 9 (0.2%) | 5 (0.1%) | 15 (0.4%) |
|  | M46.99 | 3 (0.1%) | 3 (0.1%) | 4 (0.1%) | 4 (0.1%) | 4 (0.1%) | 4 (0.1%) | 7 (0.2%) |
| M41 | M41.26 | 3 (0.1%) | 4 (0.1%) | 2 (0.1%) | 4 (0.1%) | 9 (0.2%) | 8 (0.2%) | 15 (0.4%) |


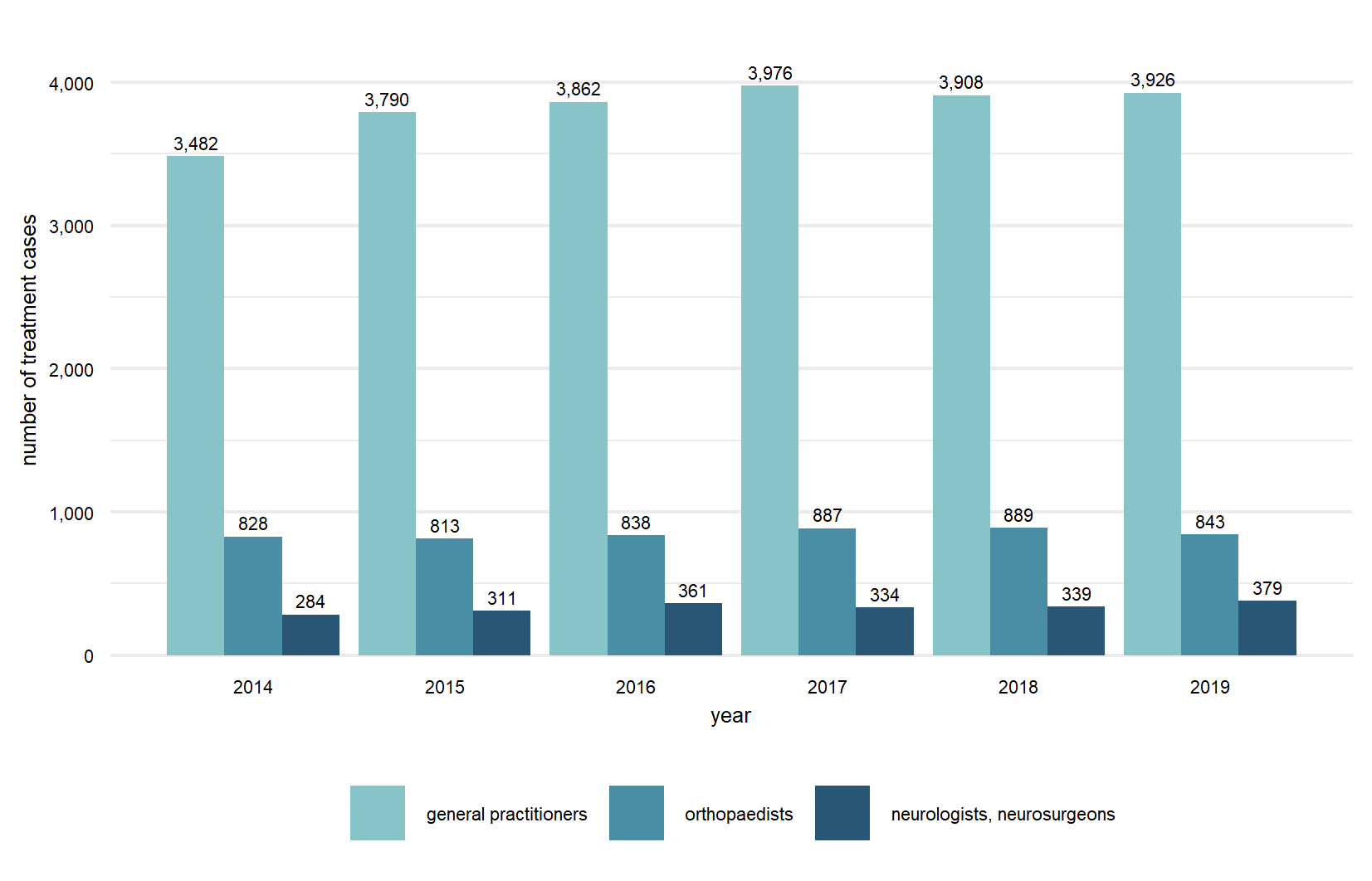


**Figure Z3:** Number of cases (i.e., billed fee schedule items with a LBP diagnosis in the same quarter) per year by treating specialist group.

**Table Z6:** Comparison of the number of patients with LBP with ICD-10 codes included in the Global Burden of Disease Study [3] to the number of patients with LBP based on the present selection of ICD-10 codes per year. During the entire observation period, 1,741 participants (70.4% of patients with LBP) received a diagnosis of the M54 category that was included in the Global Burden of Disease Study, whereas 733 participants (29.6%) received only LBP diagnoses not included in the Global Burden of Disease Study.

| Year | Number of patients with LBP diagnoses included in the Global Burden of Disease Study (M54.3, M54.4, M54.5) | Number of patients with LBP diagnoses not included in the Global Burden of Disease Study |
| --- | --- | --- |
| 2014 | 694 (48.5%) | 737 (51.5%) |
| 2015 | 729 (50.2%) | 724 (49.8%) |
| 2016 | 732 (49.8%) | 738 (50.2%) |
| 2017 | 757 (51.4%) | 716 (48.6%) |
| 2018 | 687 (48.1%) | 742 (51.9%) |
| 2019 | 662 (46.5%) | 761 (53.5%) |

[3] Ferreira ML, Luca K de, Haile LM, Steinmetz JD, Culbreth GT, Cross M, et al. Global, regional, and national burden of low back pain, 1990–2020, its attributable risk factors, and projections to 2050: a systematic analysis of the Global Burden of Disease Study 2021. The Lancet Rheumatology. 2023;5:e316–29.
